# Ocular anterior segment and corneal parameters evaluation in celiac disease

**DOI:** 10.1038/s41598-022-06058-1

**Published:** 2022-02-09

**Authors:** Maddalena De Bernardo, Livio Vitiello, Mario Gagliardi, Luigi Capasso, Nicola Rosa, Carolina Ciacci

**Affiliations:** 1 Eye Unit, Department of Medicine, Surgery and Dentistry, Scuola Medica Salernitana, University of Salerno, Salerno, Italy; 2 Celiac Centre At University Hospital San Giovanni Di Dio E Ruggi d’Aragona, University of Salerno, Salerno, Italy; 3 Corneal Transplant Unit, ASL Napoli 1, Naples, Italy

**Keywords:** Coeliac disease, Eye diseases

## Abstract

This observational case–control study evaluated the anterior ocular segment parameters of patients with celiac disease with a Scheimpflug imaging system and compared them with those of a healthy controls group, highlighting potential differences related to the underlying pathogenetic mechanisms of the disease. Seventy celiac patients and 70 healthy subjects were assessed with a comprehensive ophthalmological evaluation, including clinical history, Snellen best-corrected visual acuity, axial length (AL) measurements with IOLMaster, and anterior segment tomographic evaluation with Pentacam HR. The measurements of all keratometry values, astigmatism, steep axis, anterior and posterior Q value (asphericity), pupil diameter, pupil center, corneal apex, the thinnest point, corneal volume, anterior chamber depth from the epithelium, anterior chamber depth from endothelium, anterior chamber volume, and iridocorneal angle were also appraised. The two study groups were comparable and similar for gender, age, and AL, with no statistically significant differences regarding all analyzed tomographic parameters. Thus, ocular anterior segment parameters of celiac patients are not significantly different from those of healthy subjects, suggesting no underlying pathogenetic implications of celiac disease affecting the assessed structures. Nevertheless, a routine ophthalmological examination for all celiac patients should be recommended throughout their lifetimes due to the potential ocular manifestations of the disease.

## Introduction

Celiac disease is considered a chronic, inflammatory, systemic, and immune-mediated condition^1^, characterized by the production of autoantibodies against tissue transglutaminase, which are activated, in genetically predisposed people, by gluten and gluten-like proteins^2^.

Particularly, autoantibodies of celiac disease can bind to various extraintestinal tissues and cause different immunological diseases, such as myocarditis, dilated cardiomyopathy, epilepsy, ataxia, peripheral neuropathy, dermatitis herpetiformis, iron deficiency anemia, glomerulonephritis, liver diseases, and osteopenia^3^.

Only a few studies have shown a possible ocular involvement in celiac disease^4–6^.

The ocular involvement could be related to different mechanisms, such as accumulation of circulating immune complexes or autoantibodies in ocular tissues, cross-reactivity of cell antigenic epitopes, several vitamin deficiencies and immunogenetic factors^6^.

Some researchers have shown that the choroid of celiac patients is thicker than healthy controls^7,8^. On the other hand, concerning the anterior segment of the eye, only two studies have been previously published in the literature, showing conflicting results^9,10^.

Considering the above-mentioned potential ocular manifestations of the celiac disease, the purpose of this study is to evaluate the anterior ocular segment of celiac patients with a Scheimpflug imaging system to look for any possible tomographic signs of ocular involvement. Comparing these results with those of a healthy control group could highlight potential differences related to the underlying pathogenetic mechanisms of the disease.

## Materials and methods

### Patients selection

Adult subjects with a diagnosis of celiac disease, consecutively evaluated at the Celiac Disease Center at the Department of Medicine, Surgery, and Dentistry of the University of Salerno between September 2019 and March 2020, and a control group of healthy subjects chosen among spouses of patients and hospital staff were enrolled in this observational case–control study.

Diagnosis of celiac disease was confirmed by intestinal biopsy and serology, regardless of the time of diagnosis. Since the diagnosis, all celiac patients were under treatment with a gluten-free diet. Concerning control subjects, they had at least one negative specific serology for celiac disease and no diagnosis of any gastrointestinal diseases.

Subjects younger than 18 years of age or with systemic and ocular diseases, or patients who underwent other ophthalmic surgical procedures which could affect the anterior ocular segment^11–14^, were excluded from this study.

According to the Declaration of Helsinki's ethical principles, all participants were informed about the study's purpose, and a written informed consent was acquired. Institutional Review Board approval was also obtained from the ComEtico Campania Sud (CECS), prot. n°16544.

### Clinical examination and Scheimpflug camera imaging

A comprehensive ophthalmological evaluation, including clinical history, slit-lamp examination, Snellen best-corrected visual acuity, axial length (AL) measurements with IOLMaster (Carl Zeiss Meditec AG, Jena, Germany, version 5.4.4.0006), and tomographic evaluation with Pentacam HR (Oculus, Wetzlar, Germany) was performed.

Pentacam HR is a combined evaluation device consisting of a slit illumination system (blue led at 475 nm) and a Scheimpflug camera, which rotate together around the eye's optical axis.

Within 2 s, the device generates 50 sectional images of the corneal surface, analyzing 500 measurement points for every image (50 × 500 = 25,000 points).

During the tomographic exam, all participants were asked to sit in front of the device, with chin and forehead resting on the appropriate supports, to keep both eyes open and to fixate on a blinking fixation target in the camera’s center. The operator visualized the image of the patient’s eye on a computer screen and focused it by moving the joystick of the instrument. As soon as the image was perfectly aligned, the scan automatically started, while the participant was asked not to move and to keep eyes open.

The measurements of all keratometry values (K), astigmatism, steep axis, anterior and posterior Q value (asphericity), pupil diameter, pupil center (PC), corneal apex (CA), the thinnest point (TP), corneal volume (CV), anterior chamber depth from the epithelium (ACD_epi_), anterior chamber depth from endothelium (ACD_endo_), anterior chamber volume (ACV), and iridocorneal angle were obtained in each image.

All participants were examined between 2:00 p.m. and 3:00 p.m., and all measurements were taken in a dark room. For each participant only one eye, with at least one measurement defined as “OK” for examination quality specification by the device, was selected for the study.

### Statistical analysis

All data were analyzed with GraphPad Prism 8 (GraphPad Software, LLC, version 8.4.3). Kolmogorov–Smirnov test was performed to assess normal distribution (p > 0.05) for all data.

One-way analysis of variance was performed for all normal-distributed data, showing similar variances between the two groups (p > 0.05), except for CV. For this reason, to compare the different parameters of the two groups, two-tailed Mann–Whitney U test for not normal-distributed data, two-tailed independent samples Student *t*-test for normal-distributed data with equal variances, two-tailed independent samples Student *t*-test with Welch’s correction for normal-distributed data with unequal variances, and Chi-Square test with Yates correction for gender and astigmatism type were used. Further statistical analysis was performed by comparing male and female subjects in the two study groups, following the same above-mentioned criteria. P values less than 0.05 were considered statistically significant.

The sample size was determined by maximizing the statistical power. The analysis was performed by using G*Power software (version 3.1.9.4)^15^. A difference between two independent means (two groups) was computed. Input data were the following: α was set at 0.05; 1-β was set at 0.835; allocation ratio N2/N1 was set at 1; effect size was set as a medium at around 0.5. Results were the following: non-centrality parameter δ = 2.958; critical t = 1.977; Df = 138; sample size group 1 = 70; sample size group 2 = 70; actual power = 0.836; total sample size = 140.

## Results

Seventy patients with celiac disease and 70 healthy subjects were included, while three celiac patients with anterior segment disease (two patients with Fuchs disease and one with pterygium) and another who underwent refractive surgery were excluded. The mean disease duration of the celiac patients was 9.3 ± 8.5 years (range: 0–41 years).

The demographic characteristics of the two groups are summarized in Tables 1, 2, 3, showing no statistically significant differences for gender, age and AL between the two groups.

**Table 1:** Demographic characteristics of the two study groups.

	Celiac patients		Healthy Controls		P-value
	Mean ± SD (Range)	Median (IQ range)	Mean ± SD (Range)	Median (IQ Range)	
Patients (number)	70	–	70	–	–
Eye (number)	70	–	70	–	–
Gender (M/F)	19/51	–	25/45	–	0.36^a^
Age (years)	40.2 ± 11.4 (18.0–66.0)	41.5 (30.8–48.3)	39.8 ± 14.0 (23.0–69.0)	36.0 (26.0–53.0)	0.75^b^
AL (mm)	23.62 ± 0.96 (21.70–26.12)	23.53 (22.85–24.23)	23.84 ± 1.05 (20.82–26.11)	23.76 (23.26–24.51)	0.21^c^
Astigmatism (D)	−0.90 ± 0.70 (−3.4 to 0.5)	−0.8 (−1.4 to −0.5)	−0.90 ± 0.70 (−3.1 to 1.1)	−0.9 (−1.4 to −0.6)	0.77^b^
Astigmatism type (WTR/ATR/OBL)	58/4/8	–	58/5/7	–	0.99^a^
Age disease (years)	9.3 ± 8.5 (0–41)	7.5 (2.8–15.0)	–	–	–

^a^Chi-square test with Yates correction.

^b^Mann Whitney U test.

^c^Student t-test unpaired.

SD: Standard Deviation; IQ: interquartile; AL: Axial Length; D: Diopter; WTR: With-the-Rule; ATR: Against-the-Rule; OBL: Oblique.

**Table 2:** Demographic characteristics of the two male groups.

	Celiac males		Healthy males		P-value
	Mean ± SD (Range)	Median (IQ range)	Mean ± SD (Range)	Median (IQ Range)	
Patients (number)	19	–	25	–	–
Eye (number)	19	–	25	–	–
Age (years)	42.1 ± 13.5 (18.0–66.0)	44.0 (34.0–51.0)	45.7 ± 12.8 (25.0–63.0)	50.0 (30.0–56.5)	0.18^b^
AL (mm)	23.64 ± 0.76 (22.42–24.85)	23.74 (22.83–24.24)	23.97 ± 0.95 (22.42–26.11)	23.83 (23.43–24.42)	0.22^c^
Astigmatism (D)	−0.90 ± 1.00 (−3.4 to 0.5)	−0.6 (−1.4 to −0.2)	−0.72 ± 0.78 (−2.2 to 1.0)	−0.9 (−1.3 to −0.1)	0.52^c^
Astigmatism type (WTR/ATR/OBL)	12/4/3	–	17/3/5	–	0.98^a^

^a^Chi-square test with Yates correction.

^b^Mann Whitney U test.

^c^Student t-test unpaired.

SD: Standard Deviation; IQ: interquartile; AL: Axial Length; D: Diopter; WTR: With-the-Rule; ATR: Against-the-Rule; OBL: Oblique.

**Table 3:** Demographic characteristics of the two female groups.

	Celiac females		Healthy females		P-value
	Mean ± SD (Range)	Median (IQ range)	Mean ± SD (Range)	Median (IQ Range)	
Patients (number)	51	–	45	–	–
Eye (number)	51	–	45	–	–
Age (years)	39.5 ± 10.6 (21.0–58.0)	39.0 (30.0–48.0)	36.5 ± 13.6 (23.0–69.0)	32.0 (25.0–48.5)	0.08^b^
AL (mm)	23.61 ± 1.04 (21.70–26.12)	23.49 (22.86–24.23)	23.76 ± 1.11 (20.82–25.77)	23.72 (23.13–24.67)	0.50^c^
Astigmatism (D)	−0.96 ± 0.57 (−2.9 to 0.4)	−0.8 (−1.4 to −0.6)	−1.01 ± 0.72 (−3.1 to 1.1)	−1.0 (−1.5 to −0.6)	0.60^b^
Astigmatism type (WTR/ATR/OBL)	46/0/5	–	41/2/2	–	0.57^a^

^a^Chi-square test with Yates correction.

^b^Mann Whitney U test.

^c^Student t-test unpaired.

SD: Standard Deviation; IQ: interquartile; AL: Axial Length; D: Diopter; WTR: With-the-Rule; ATR: Against-the-Rule; OBL: Oblique.

Concerning slit-lamp examination, no clinical signs of corneal damage were found in the included celiac patients.

Regarding all analyzed tomographic parameters, no statistically significant differences were found between the two studied groups, as summarized in Table 4. The same results were obtained by comparing males and females between the two groups, as shown in Tables 5, 6.

**Table 4:** Tomographic parameters assessed with Pentacam HR in the two study groups.

	Celiac patients		Healthy controls		P-value
	Mean ± SD (Range)	Median (IQ Range)	Mean ± SD (Range)	Median (IQ Range)	
K_1_ front (D)	43.1 ± 1.3 (40.0–47.0)	43.0 (42.4–43.6)	43.3 ± 1.5 (40.8–47.5)	43.2 (42.1–44.2)	0.56^a^
K_2_ front (D)	44.1 ± 1.4 (40.7–48.2)	43.9 (43.2–44.8)	44.3 ± 1.5 (41.3–48.8)	44.3 (43.1–45.2)	0.33^a^
K_mean_ front (D)	43.6 ± 1.3 (40.4–47.4)	43.6 (42.8–44.3)	43.8 ± 1.5 (41.1–48.2)	43.9 (42.5–44.7)	0.39^a^
K_max_ (D)	44.7 ± 1.5 (41.1–48.7)	44.3 (43.7–45.6)	44.8 ± 1.5 (41.7–49.1)	44.9 (43.7–45.8)	0.40^a^
K_1_ back (D)	−6.1 ± 0.2 (−6.7 to −5.6)	−6.1 (−6.3 to −6.0)	−6.2 ± 0.3 (−7.0 to −5.6)	−6.2 (−6.3 to −6.0)	0.82^a^
K_2_ back (D)	−6.5 ± 0.2 (−7.2 to −5.9)	−6.4 (−6.6 to −6.3)	−6.5 ± 0.3 (−7.3 to −5.9)	−6.5 (−6.6 to −6.2)	0.92^a^
Q-value front	−0.32 ± 0.11 (−0.62 to −0.02)	−0.31 (−0.38 to −0.25)	−0.32 ± 0.14 (−0.73 to −0.07)	−0.31 (−0.39 to −0.23)	0.94^b^
Q-value back	−0.36 ± 0.14 (−0.83 to −0.10)	−0.36 (−0.43 to −0.26)	−0.35 ± 0.14 (−0.64 to −0.09)	−0.34 (−0.46 to −0.22)	0.55^b^
PD (mm)	3.04 ± 0.54 (2.05–4.49)	3.01 (2.69–3.32)	3.09 ± 0.54 (2.26–4.58)	3.02 (2.71–3.48)	0.72^a^
PC (μm)	542.2 ± 32.9 (475.0–643.0)	536.0 (520.3–560.3)	538.7 ± 32.1 (434.0–603.0)	541.5 (514.0–561.3)	0.77^a^
CA (μm)	543.2 ± 32.3 (477.0–645.0)	537.5 (522.8–560.3)	539.8 ± 32.4 (438.0–614.0)	541.0 (514.0–563.5)	0.53^b^
TP (μm)	537.2 ± 32.7 (471.0–642.0)	532.5 (514.8–557.0)	533.0 ± 32.1 (432.0–603.0)	537.0 (507.0–557.0)	0.54^b^
CV (mm^3^)	60.6 ± 3.2 (53.8–67.9)	60.4 (58.6–62.5)	60.3 ± 4.4 (50.2–69.7)	59.6 (57.5–63.1)	0.72^c^
ACD_epi_ (mm)	3.40 ± 0.34 (2.48–4.08)	3.42 (3.17–3.62)	3.49 ± 0.37 (2.62–4.27)	3.51 (3.20–3.76)	0.15^b^
ACD_endo_ (mm)	2.86 ± 0.34 (1.97–3.51)	2.86 (2.63–3.08)	2.95 ± 0.37 (2.00–3.76)	2.94 (2.68–3.24)	0.14^b^
ACV (mm^3^)	160.7 ± 35.4 (84.0–240.0)	160.0 (134.8–186.3)	168.4 ± 40.4 (82.0–249.0)	166.0 (132.8–193.3)	0.24^b^
ICA (degrees)	35.0 ± 5.8 (21.6–48.6)	35.0 (31.5–39.5)	35.6 ± 5.9 (21.3–48.0)	36.1 (31.2–39.6)	0.51^b^

^a^Mann Whitney U test.

^b^Student t-test unpaired.

^c^Student t-test unpaired with Welch’s correction.

SD: Standard Deviation; IQ: Interquartile; D: Diopter; PD: Pupil Diameter; PC: Pupil Center; CA: Corneal Apex; TP: Thinnest Point; CV: Corneal Volume; ACD_epi_: Anterior Chamber Depth from epithelium; ACD_endo_: Anterior Chamber Depth from endothelium; ACV: Anterior Chamber Volume; ICA: Iridocorneal Angle.

**Table 5:** Tomographic parameters assessed with Pentacam HR in the two male groups.

	Celiac males		Healthy males		P-value
	Mean ± SD (Range)	Median (IQ Range)	Mean ± SD (Range)	Median (IQ Range)	
K_1_ front (D)	43.1 ± 1.3 (41.3–46.5)	42.8 (42.3–43.5)	43.4 ± 1.7 (40.9–47.5)	43.5 (42.0–44.2)	0.56^a^
K_2_ front (D)	44.1 ± 1.4 (41.8–47.9)	43.8 (43.0–45.3)	44.3 ± 1.7 (41.3–48.8)	44.3 (42.9–45.0)	0.71^a^
K_mean_ front (D)	43.6 ± 1.3 (41.7–47.2)	43.4 (42.6–44.5)	43.9 ± 1.7 (41.1–48.2)	43.9 (42.6–44.5)	0.62^a^
K_max_ (D)	44.7 ± 1.4 (43.0–48.4)	44.4 (43.4–45.9)	45.0 ± 1.6 (42.6–49.1)	44.9 (43.9–45.6)	0.60^a^
K_1_ back (D)	−6.1 ± 0.2 (−6.7 to −5.8)	−6.1 (−6.2 to −6.0)	−6.2 ± 0.3 (−7.0 to −5.6)	−6.2 (−6.4 to −5.9)	0.65^a^
K_2_ back (D)	−6.5 ± 0.2 (−7.1 to −6.2)	−6.5 (−6.6 to −6.3)	−6.5 ± 0.4 (−7.3 to −5.9)	−6.4 (−6.7 to −6.2)	0.99^a^
Q-value front	−0.33 ± 0.14 (−0.52 to −0.02)	−0.32 (−0.46 to −0.23)	−0.31 ± 0.18 (−0.73 to −0.09)	−0.26 (−0.43 to −0.18)	0.80^a^
Q-value back	−0.41 ± 0.18 (−0.83 to −0.12)	−0.37 (−0.49 to −0.30)	−0.36 ± 0.16 (−0.64 to −0.09)	−0.31 (−0.52 to −0.22)	0.32^b^
PD (mm)	2.86 ± 0.68 (2.05–4.49)	2.70 (2.47–3.19)	2.93 ± 0.53 (2.26–4.47)	2.73 (2.53–3.34)	0.39^b^
PC (μm)	540.1 ± 30.9 (475.0–598.0)	540.0 (524.0–555.0)	540.5 ± 35.4 (434.0–589.0)	545.0 (516.0–566.5)	0.97^a^
CA (μm)	541.9 ± 30.5 (477.0–601.0)	542.0 (528.0–556.0)	541.8 ± 35.2 (438.0–588.0)	548.0 (515.5–567.0)	0.99^a^
TP (μm)	534.1 ± 30.6 (471.0–593.0)	534.0 (514.0–553.0)	535.1 ± 35.0 (432.0–582.0)	539.0 (506.5–560.0)	0.92^a^
CV (mm^3^)	60.2 ± 2.9 (53.8–64.4)	60.3 (58.7–62.4)	60.6 ± 4.9 (50.5–69.7)	59.5 (58.0–64.8)	0.73^a^
ACD_epi_ (mm)	3.45 ± 0.41 (2.72–4.08)	3.47 (3.12–3.92)	3.47 ± 0.42 (2.67–4.27)	3.42 (3.16–3.82)	0.91^a^
ACD_endo_ (mm)	2.91 ± 0.41 (2.22–3.51)	2.95 (2.58–3.39)	2.93 ± 0.42 (2.16–3.76)	2.90 (2.61–3.30)	0.92^a^
ACV (mm^3^)	163.5 ± 41.5 (106.0–240.0)	160.0 (132.0–187.0)	168.5 ± 44.4 (92.0–246.0)	170.0 (126.5–206.0)	0.70^a^
ICA (degrees)	34.5 ± 6.1 (21.7–42.8)	35.2 (29.6–39.6)	34.7 ± 6.5 (22.2–47.6)	35.1 (30.2–39.0)	0.91^a^

^a^Student t-test unpaired.

^b^Mann Whitney U test.

SD: Standard Deviation; IQ: Interquartile; D: Diopter; PD: Pupil Diameter; PC: Pupil Center; CA: Corneal Apex; TP: Thinnest Point; CV: Corneal Volume; ACD_epi_: Anterior Chamber Depth from epithelium; ACD_endo_: Anterior Chamber Depth from endothelium; ACV: Anterior Chamber Volume; ICA: Iridocorneal Angle.

**Table 6:** Tomographic parameters assessed with Pentacam HR in the two female groups.

	Celiac females		Healthy females		P-value
	Mean ± SD (Range)	Median (IQ Range)	Mean ± SD (Range)	Median (IQ Range)	
K_1_ front (D)	43.2 ± 1.3 (40.0–47.0)	43.0 (42.5–43.8)	43.2 ± 1.4 (40.8–45.8)	43.0 (42.1–44.4)	0.83^a^
K_2_ front (D)	44.1 ± 1.5 (40.7–48.4)	43.9 (43.2–44.8)	44.3 ± 1.4 (41.4–46.6)	44.4 (43.2–45.3)	0.63^b^
K_mean_ front (D)	43.7 ± 1.4 (40.4–47.4)	43.6 (42.9–44.3)	43.8 ± 1.4 (41.2–46.2)	43.9 (42.5–44.8)	0.47^a^
K_max_ (D)	44.7 ± 1.5 (41.1–48.7)	44.3 (43.7–45.6)	44.8 ± 1.4 (41.7–46.9)	44.9 (43.7–45.8)	0.77^b^
K_1_ back (D)	−6.1 ± 0.2 (−6.7 to −5.6)	−6.1 (−6.3 to −6.0)	−6.2 ± 0.3 (−6.7 to −5.7)	−6.1 (−6.3 to −6.0)	0.91^a^
K_2_ back (D)	−6.5 ± 0.3 (−7.2 to −5.9)	−6.4 (−6.6 to −6.3)	−6.5 ± 0.3 (−6.9 to −5.9)	−6.5 (−6.6 to −6.2)	0.77^a^
Q-value front	−0.32 ± 0.11 (−0.62 to −0.06)	−0.30 (−0.38 to −0.25)	−0.32 ± 0.11 (−0.63 to −0.07)	−0.32 (−0.39 to −0.25)	0.71^b^
Q-value back	−0.34 ± 0.12 (−0.67 to −0.10)	−0.33 (−0.42 to −0.25)	−0.34 ± 0.13 (−0.64 to −0.11)	−0.34 (−0.45 to −0.22)	0.93^b^
PD (mm)	3.11 ± 0.47 (2.11–4.43)	3.13 (2.79–3.33)	3.18 ± 0.53 (2.26–4.58)	3.10 (2.79–3.51)	0.53^b^
PC (μm)	543.0 ± 33.9 (477.0–643.0)	536.0 (518.0–562.0)	537.7 ± 30.4 (469.0–603.0)	538.0 (514.0–559.5)	0.52^a^
CA (μm)	543.7 ± 33.3 (477.0–645.0)	537.0 (521.0–561.0)	538.6 ± 31.2 (472.0–614.0)	537.0 (514.0–560.5)	0.50^a^
TP (μm)	538.3 ± 33.6 (474.0–642.0)	532.0 (515.0–557.0)	533.0 ± 30.7 (466.0–603.0)	536.0 (509.0–553.5)	0.55^a^
CV (mm^3^)	60.7 ± 3.4 (54.0–67.9)	60.5 (58.1–63.8)	60.2 ± 4.2 (50.2–69.2)	59.7 (57.2–62.9)	0.49^b^
ACD_epi_ (mm)	3.38 ± 0.32 (2.48–4.06)	3.34 (3.22–3.62)	3.50 ± 0.34 (2.62–4.12)	3.52 (3.21–3.74)	0.09^b^
ACD_endo_ (mm)	2.84 ± 0.31 (1.97–3.44)	2.79 (2.67–3.06)	2.96 ± 0.35 (2.00–3.64)	3.00 (2.72–3.19)	0.07^b^
ACV (mm^3^)	159.7 ± 33.3 (84.0–240.0)	160.0 (135.0–186.0)	168.3 ± 38.5 (82.0–249.0)	161.0 (141.5–188.5)	0.24^b^
ICA (degrees)	35.1 ± 5.8 (21.6–48.6)	34.9 (31.9–39.5)	36.1 ± 5.6 (21.3–48.0)	36.5 (32.7–40.5)	0.41^b^

^a^Mann Whitney U test.

^b^Student t-test unpaired.

SD: Standard Deviation; IQ: Interquartile; D: Diopter; PD: Pupil Diameter; PC: Pupil Center; CA: Corneal Apex; TP: Thinnest Point; CV: Corneal Volume; ACD_epi_: Anterior Chamber Depth from epithelium; ACD_endo_: Anterior Chamber Depth from endothelium; ACV: Anterior Chamber Volume; ICA: Iridocorneal Angle.

## Discussion

Celiac disease is a systemically involved autoimmune condition that primarily affects the small intestine but could also exhibit multiple extraintestinal symptoms^3^. Among these, the eye definitely represents one of the disease's target organs, and cataract, uveitis, dry eye, neuro-ophthalmic manifestations, night blindness, occlusion of the central retinal vein, and orbitopathy associated with thyroid can occur^16^.

The present study is the largest one comparing the ocular anterior segment of celiac patients to a control healthy group, with the purpose to point out potential differences that could be explained by the underlying pathogenetic mechanisms of the celiac disease.

However, no statistically significant differences were found in this study for any of the parameters tomographically assessed.

The results of the present study are in contrast with that ones provided by two previous studies published in the literature^9,10^.

Karatepe Hashas et al.^9^ utilized the Pentacam system to appraise 31 celiac children and 34 controls (62 eyes and 68 eyes, respectively), revealing ACD and ACV of celiac patients to be significantly smaller than control subjects. The authors tried to explain these findings with the auto-antibodies affinity to trabecular network, suggesting further pathophysiological studies to verify their hypothesis.

Inversely, Hazar et al.^10^ using the Sirius system to evaluate 31 adult celiac patients and 25 healthy controls (58 eyes and 50 eyes, respectively), found ACD and iridocorneal angle of celiac patients to be significantly larger than healthy subjects, while no significant difference for ACV was found. Even in this case, the authors tried to explain their results with the autoantibodies affinity to anterior segment structures, as they also found a positive correlation between ACV and anti-gliadin IgA. Furthermore, they also hypothesized that their findings could be due to the autoantibodies or circulating immune complexes deposition in the eye tissue, suggesting to perform further long-term follow-up studies.

Several explanations could be adduced to try to clarify the differences between these two studies and with the present one^9,10^.

First of all, the present study has been carried out on a larger sample size, which was determined with the power calculation evaluation^15^. For this reason, previous studies^9,10^ may have provided statistically significant results, conflicting with each other, due to a not large and significant enough sample size.

Moreover, the present study examined only one eye for each participant, while both the previous studies^9,10^ assessed both eyes in some patients and in some others only one eye. This could create a potential statistical bias which could alter the results, as discussed by McAlinden et al.^17,18^.

Besides, the present study evaluated two different ACD measurements; ACD*epi*, which is the ACD measured from the corneal epithelium, and ACD*endo*, that is the ACD measured from the corneal endothelium. However, no statistically significant difference was found in the present study between the two study groups for these parameters, while the previous studies^9,10^ showed a contrasting statistically significant difference without specifying which ACD was evaluated.

Another difference with the previously published studies could be the difference in the age of the appraised groups. In fact, Karatepe Hashas et al.^9^ and Hazar et al.^10^ evaluated participants with a mean age of approximately 30 and 10 years respectively younger than those of this study. Ocular anterior segment structures have been shown to change with age^19–21^, but in our opinion this should not explain the differences, both because celiac and control groups were in the same age range, and because potential differences should be related to the progression of the disease, and not be present in the early stages of life.

Finally, Hazar et al.^10^ utilized a different Scheimpflug device (Sirius) in their study. It has been proven that Scheimpflug devices utilize slightly different measurement algorithms^22,23^ and maybe this could account for the differences between the two studies.

A limitation of this study could be the evaluation of only celiac patients under treatment with a gluten-free diet. Further studies comparing potential differences between treated and untreated celiac patients could be of interest to better identify the impact of the gluten-free diet on possible ocular modifications.

In conclusion, the ocular anterior segment parameters of celiac patients are not significantly different from those of healthy subjects, suggesting none of the underlying pathogenetic implications of this disease affects the assessed structures. Nevertheless, due to the association between celiac disease and other ocular disorders, such as cataract, uveitis, dry eye, neuro-ophthalmic manifestations, night blindness, occlusion of the central retinal vein, and orbitopathy associated with thyroid, a routine ophthalmological examination for all celiac patients should be recommended throughout their lifetimes.

## Data Availability

The datasets generated and analyzed during the current study are available from the corresponding author on reasonable request. GPower: https://www.psychologie.hhu.de/arbeitsgruppen/allgemeine-psychologie-und-arbeitspsychologie/gpower.html
